# Response to Joep M. A. Lange

**DOI:** 10.1371/journal.pmed.0020347

**Published:** 2005-10-25

**Authors:** Melissa Ditmore

**Affiliations:** **1**Research for Sex Work, Chiang Mai, Thailand; Network of Sex Work Projects, Hong Kong, China

Joep M. A. Lange, in his *PLoS Medicine* Perspective [1], neglected the complaints of the sex workers in the tenofovir trials—a lack of attention to their long-term health care, an appalling lack of answers to their questions about side effects, inadequate translation of the trial materials in some places, and, in past experience, a failure to deliver any new drugs to sex workers in the developing world once a trial is complete. Cambodian sex workers were offended by the assertion of trial conductors that they should be the bodies on which tests are conducted for the benefit of the rest of the world, without guarantees of health care for side effects and infections that occur during a trial, and without even receiving answers to their questions about the trial. Most people in developed countries would have been offended if they'd been asked to do the same. The expectation that marginalized populations will accept such ungracious treatment is patently offensive.

Sex workers would like to see research continue, and would like even more to see sex workers have access to effective treatment and prevention. Trial participants in developed countries have been motivated to push for faster development of drugs by the need for treatment. Sex workers have met with the organizers and supporters of the trials—this is extremely cooperative! Some meetings have been good and others have been baldfaced tokenism, some even without language translation. Nothing says “we don't care what you have to say” louder than not translating for someone flown thousands of miles to attend a meeting for two days.

When sex workers and other marginalized people are genuine participants with input at all stages of research, they will be eager research participants. There is a good example of this in the journal *Research for Sex Work*. There is an update on the tenofovir trials, but the lead article is titled “Cambodian sex workers conduct their own research” [2]. These sex workers were invited to choose a topic and design research that would be useful for them. They did so because they had input into the research at every level. Drug researchers should take note.
